# Multiple Reassortments, Limited Geographic Spread, and Comparable Pathogenicity of Crimean‐Congo Hemorrhagic Fever Viruses in Sivas Province, Türkiye

**DOI:** 10.1002/jmv.71027

**Published:** 2026-06-22

**Authors:** Qianying Lin, Nazif Elaldi, Mesut Yigit, Baris Yildiz, Roger Hewson, Martín López‐García, Grant Lythe, Ayse Nur Pektas, Binnur Koksal, Tuba Nur Tasseten, Ahmet Deniz, Hilal Bedir, Yasemin Cakir Kiymaz, Zati Vatansever, Carmen Molina‐París, Thomas Leitner

**Affiliations:** ^1^ Theoretical Biology and Biophysics Los Alamos National Laboratory Los Alamos NM USA; ^2^ Division of Biostatistics The Ohio State University Columbus OH USA; ^3^ Department of Infectious Diseases and Clinical Microbiology Cumhuriyet University Sivas Turkiye; ^4^ Department of Parasitology Kafkas University Kars Turkiye; ^5^ Life Sciences and Technology Application and Research Center Kafkas University Kars Turkiye; ^6^ Virology and Pathogenesis UKHSA Porton Down Salisbury UK; ^7^ Department of Infection Biology London School of Hygiene and Tropical Medicine London UK; ^8^ Wellcome Sanger Institute Hinxton UK; ^9^ School of Mathematics University of Leeds Leeds UK; ^10^ Cumhuriyet University Advanced Technology Application and Research Center (CUTAM) Cumhuriyet University Sivas Turkiye; ^11^ Department of Nutrition and Dietetics Cumhuriyet University Sivas Turkiye; ^12^ Department of Molecular Biology and Genetics Cumhuriyet University Sivas Turkiye; ^13^ Department of Parasitology, Faculty of Medicine Kafkas University Kars Turkiye

## Abstract

Crimean‐Congo hemorrhagic fever virus (CCHFV) is a segmented RNA virus that can cause severe hemorrhagic fever and is primarily transmitted to humans and other mammals through tick bites. Since segment reassortment can increase genetic diversity and disease severity, we sampled infected ticks and humans in Sivas Province, Türkiye, a region with high CCHFV prevalence. Analysis of 113 human and 23 Hyalomma tick CCHFV samples revealed three main phylogenetic clades in Sivas. The most common clade (SIVAS‐2) was involved in multiple reassortants. Phylogenetic analysis indicated multiple independent reassortment events involving all three CCHFV genome segments. The most common reassortant (1.1.2) was detected only in the Zara district of Sivas in both humans and ticks. We found that most infections were geographically limited to local spread and that infections with more than one variant and reassortment were exceptional. We found no significant clinical differences between human SIVAS‐1, SIVAS‐2 and the 1.1.2 reassortant infections. Thus, within the limits of the available sample size, we found no evidence that the two parental forms differed in disease severity or that the 1.1.2 reassortant was associated with increased severity. Continued CCHFV surveillance could uncover future forms of the virus with altered characteristics.

## 1 Introduction

Crimean‐Congo hemorrhagic fever virus (CCHFV; family *Nairoviridae*) is a tick‐borne pathogen with the broadest geographic range of any tick‐borne virus, spanning Africa, Asia, the Middle East, and southern Europe [1, 2, 3]. The geographic range has expanded over the past 25 years, with particularly high incidence in Türkiye and the Middle East, and the recent spread into southern Europe is likely to have originated in Türkiye [3, 4, 5, 6, 7].

CCHFV is transmitted between ticks and vertebrate hosts, including humans, primarily through tick bites from *Hyalomma marginatum* or through contact with body fluids from infected animals or humans [8]. The disease severity of CCHFV human infections varies largely from asymptomatic to mortality in the 5% to 80% range [3]. The most severe cases appear to be associated with outbreaks such as the 1944 Crimean outbreak and later outbreaks in Kosovo and Türkiye [9]. In 2024, several confirmed CCHF cases and at least one fatality were reported in Spain, and one fatality was reported in Portugal, underscoring its expansion as a global public health threat [10].

CCHFV is a segmented RNA virus with a tri‐partite RNA genome, consisting of small (S), medium (M), and large (L) segments, which encode the nucleoprotein, glycoproteins, and RNA polymerase, respectively [1, 11]. These genomic segments display the highest level of genetic diversity among all arboviruses, with more than 20% genetic differences [12], and multiple genetically divergent phylogenetic clades exist within each segment [7, 12, 13]. Specifically, M segment divergence within some African/Asian clades could exceed 30–35% [14]. Importantly, these genomic segments may reassort when hosts are co‐infected with multiple distinct viral strains, creating new viral combinations throughout CCHFV's evolutionary history [11, 15]. Recent next‐generation sequencing (NGS) studies have demonstrated high genetic variability, particularly in the M segment, highlighting reassortment's critical role in CCHFV evolution [16].

In Türkiye, CCHFV is a major cause of zoonotic disease outbreaks, with recurrent human cases reported since its recognition in 2002 and a marked concentration in rural areas of Anatolia [17, 18, 19]. The combination of favorable ecological conditions for *Hyalomma* ticks and extensive agricultural and livestock activities has raised significant public health concerns [20, 21, 22]. Molecular studies have shown that Turkish CCHFV strains largely cluster within the Europe lineage, while broader CCHFV genomic studies show substantial genetic diversity, long‐distance viral movement, and repeated reassortment among the S, M, and L segments [11, 14, 23, 24, 25, 26]. These evolutionary patterns can complicate phylogenetic interpretation and molecular surveillance, making it important to characterize segment‐level diversity and reassortment when assessing outbreak risk, diagnostic robustness, and potential differences in pathogeneicity [15, 27].

Genetic studies from Türkiye have characterized the S, M, and L segments and documented co‐circulating lineages with frequent segment reassortment; reports of within‐segment recombination also exist, though findings are mixed [2, 3, 23, 28]. However, the relationship between viral genotype and clinical outcomes, including disease severity, viremia levels, and mortality, remains poorly understood due to limited studies linking phylogenetic data to clinical parameters [29].

To assess the frequency of geographically co‐circulating genetic variants and the risk of reassortment generating novel phenotypes, we sampled 113 human and 23 *Hyalomma* tick infections in Sivas, a high‐prevalence region in Türkiye, between 2011 and 2023. In addition, the impact of CCHFV phylogenetic clusters on disease severity and case‐fatality rate was investigated. We found that both rare and common local forms reassorted; however, no increase in disease severity was observed among the prevalent genetic or reassortant forms in humans during this time period.

## 2 Methods

### 2.1 Study Design, Ethics Statement, and Human Participants

The human participants in this study were part of either (1) a prospective study conducted between 2022 and 2023 or (2) a retrospective study conducted between 2011 and 2021, both run by the Department of Infectious Diseases and Clinical Microbiology, Faculty of Medicine, Sivas Cumhuriyet University (SCU), Sivas, Türkiye. Ethical approval was obtained from the Ankara City Hospital Clinical Research Ethics Committee on the 08th September 2021, with approval number E1/2015/2021. The study was conducted in accordance with good clinical practice principles and the Declaration of Helsinki. Signed informed consent (IC) was obtained from all patients enrolled in the prospective follow‐up study. On admission, three sets of whole venous blood samples (in total, 6‐8 mL) were collected by venepuncture from CCHF‐suspected patients [30]. One set was sent for routine laboratory analyses, one set was used for CCHFV‐specific RT‐PCR analysis, and another set was stored at $-80^{\circ }\;C\;$ after separation of serum for possible NGS. The diagnosis of CCHF was confirmed by commercial real‐time RT‐PCR test kits (Altona Diagnostics, Hamburg, Germany, or CCHFVD01100, Bioeksen R&D Technologies, Türkiye) at the reference laboratory of the Turkish Ministry of Health, General Directorate of Public Health, Ankara, Türkiye. The patients were all hospitalized in the adult clinic of the Department of Infectious Diseases and Clinical Microbiology (IDCM) at Sivas Cumhuriyet University Hospital (SCUH), Sivas, Türkiye. They were followed by expert clinicians, nurses, and healthcare workers until discharge or death. During hospitalization, they were evaluated daily for standard medical care needs, including intravenous fluid support, fresh frozen plasma, erythrocyte suspension, and platelet suspension [30]. On the day of admission, complete blood count (CBC), aspartate aminotransferase (AST), alanine aminotransferase (ALT), lactate dehydrogenase (LDH), creatine phosphokinase (CPK), and coagulation panel, i.e., prothrombin time (PT), activated partial thromboplastin time (aPTT), plasma fibrinogen, and D‐dimer concentrations, were measured as part of routine daily laboratory tests and repeated as needed. The CCHF patients were grouped as follows: (i) low risk (0–4), (ii) intermediate risk (5–9), and (iii) high risk (10–16), based on the patients' acute stage of Severity Grading System Score (SGS Score) and the corresponding risk level (SGS Risk), which SCUH doctors calculated on the hospitalization day [31]. The Disseminated Intravascular Coagulation Score (DIC Score) was also included [32]. Patients were transferred to the intensive care unit (ICU) when their clinical situation worsened. The epidemiological data, including age, sex, location (village/county/region), occupation, the dates of symptoms onset, hospitalization, patient samplings, and discharge/death dates, clinical data including main symptoms and signs, daily routine laboratory test results, and disease outcome data (fatal or non‐fatal), were retrieved from the electronic patient files for each subject.

### 2.2 Tick Samples

Tick samples were collected between 2022 and 2023 from various habitats within Sivas Province. Sampling locations were selected based on previous field observations and on rural residence data for patients admitted to the IDCM at SCUH. Ticks were collected from two sources: from the ground as unfed adults seeking a host and from feeding adults on cattle. Ticks collected from animals were immediately placed in field freezers. In contrast, ticks collected from the ground were kept alive at room temperature throughout the field study and subsequently transported to the Tick Research Unit of the Department of Parasitology, Faculty of Veterinary Medicine, Kafkas University, Kars, Türkiye.

### 2.3 CCHFV Extraction From Ticks and Humans and Sequencing

For tick samples, after species‐level identification [33], the ticks were cataloged individually and stored at $-80^{\circ }\;C\;$. Total RNA extraction from ticks was performed using the *BlackPREP Tick DNA/RNA Kit* (Innuscreen, Germany), which includes ceramic‐bead‐based homogenization followed by RNA and DNA isolation. Cooling blocks were used throughout all homogenization procedures to prevent the samples from reaching room temperature. All steps of the extraction process were carried out according to the manufacturer's instructions. To detect CCHFV in the extracted RNA samples, quantitative RT‐PCR was performed. Gene amplification was conducted using the TaqMan Fast Virus 1‐Step Master Mix (Thermo Fisher Scientific, USA). At the same time, primer/probe sequences and PCR conditions followed the protocol described in D'Addiego et al. [34]. Samples with Ct ≤ 38 were subjected to sequencing. Sequencing was performed through an outsourced service (Gen‐Era Diagnostics, Türkiye). Illumina sequencing libraries were prepared using the Nextera XT v2 kit (Cat. No. FC‐131‐1096, Illumina) and sequenced on a 2 × 150 bp paired‐end Illumina MiSeq platform operated by Gen‐Era Diagnostics. All libraries were quantified using the Qubit dsDNA HS assay (Thermo Fisher Scientific, USA) and the Bioanalyzer HS DNA assay (Agilent), and normalized to 4 nM with molecular‐grade water. Libraries were then pooled, denatured in freshly prepared 0.2 N NaOH for 5 min, and diluted to a final concentration of 8 pM using the HT1 buffer provided with the MiSeq Reagent Kit v2 Micro (Illumina, USA).

For human samples, serum samples collected from CCHFV RT‐PCR positive cases were thawed at room temperature in a containment level (CL) 2 safety cabinet; briefly, 140 μl of each sample was added to 560 μl of buffer AVL and 560 μl of ethanol. RNA was extracted utilizing the QIAamp Viral RNA Mini Kit (Cat. No. 52904, Qiagen) following the manufacturer's instructions. RNA was eluted in 60 μl of nuclease‐free water and stored at $-80^{\circ }\;C\;$. After the completion of RNA extraction from patient serum, the RNA samples were processed by commercial quantitative real‐time RT‐PCR test kits (CCHFVD01100, Bioeksen R&D Technologies, Türkiye). The real‐time RT‐PCR assay was performed using the StepOnePlus^TM^ Real‐Time PCR system (Applied Biosystems, Foster City, CA, USA). Samples with Ct ≤ 28 were subjected to sequencing.

### 2.4 Bioinformatic Assembly and Consensus Generation

We developed a comprehensive bioinformatic pipeline to process and analyze CCHFV genome data from 113 human and 23 tick samples. Before processing the samples, we constructed reference genome collections for each viral segment (S, M, and L) by retrieving all CCHFV sequences with lengths between 1400 and 2000 for S, 5000 and 6000 for M, and 10,000 and 15,000 for L, from Genbank [35], resulting in 524, 383, and 401 sequences respectively as of February 25, 2026, for this study.

We processed our 136 samples using sequencing‐method‐specific approaches. For 124 samples (101 human, 23 tick) prepared using standard NGS protocols, we performed *de novo* assembly with SPAdes and quality assessment with QUAST [36, 37]. Raw reads were then mapped to custom reference sequences using shiver, a mapping tool initially developed for HIV but generalizable to other viruses, to generate consensus sequences [38]. The remaining 12 human samples, prepared using Sequence‐Independent, Single‐Primer Amplification (SISPA), were processed with QuasiBAM for direct consensus sequence generation [39].

The resulting consensus sequences were filtered based on insertion‐deletion frequency derived from all collected GenBank reference sequences, and this filtering was applied separately for each virus segment. As a result, we retained S and M consensus sequences with no indels and L consensus sequences with no more than one indel, to minimize inclusion of sequences with probable assembly or alignment artefacts while allowing for the greater length and complexity of the L segment. The remaining sequences were then aligned with carefully selected reference sequences using MAFFT [40], followed by trimming procedures to eliminate poorly aligned regions and ensure consistency across the three viral segments. The resulting alignments were then used to select the 10 closest GenBank reference segment sequences to each of our consensus sequences for the segment trees phylogenetic assessment. A final standardization step established a unified set of reference sequences that are shared across all segments, facilitating comprehensive comparative analyses. After filtering and quality control, we retained 102 sequences (83 human, 19 tick) for the S segment, 90 sequences (71 human, 19 tick) for the M segment, and 104 sequences (87 human, 17 tick) for the L segment, along with 33 shared reference sequences for each segment. The resulting high‐quality consensus sequences, together with their metadata, have been deposited in GenBank under accession numbers PX874843‐PX875138.

### 2.5 Phylogenetic Reconstruction and Recombination Detection

Phylogenetic trees were reconstructed for each segment using PhyML with the GTR+$\Gamma_{4}$ substitution model with BioNJ starting tree and 100 bootstrap replicates [41], and visualized using the R package ggtree [42]. Primary and major full‐genome references were also added for a comprehensive view by reconstructing a full‐genome *master tree*, including AP92, TADJ_HU8966, IbAr10200, and a set of recent Armenian sequences [11, 25, 43]. To assess potential within‐segment recombination in our data, we performed recombination analysis on our Sivas sequences using the Recombination Identification Program (RIP) [44], which calculates the similarity (i.e., the fraction of matching characters) of a query sequence to potential ancestral sequences in a sliding window analysis. We used CCHFV reference sequences as potential ancestral representatives selected to represent the major phylogenetic clades found in this study (SIVAS‐1, SIVAS‐2, SIVAS‐3, Russia, and Kosovo) [24, 25, 45, 46, 47]. Recombination would be detected when different portions of a sequence show the highest similarity to different reference clades. In addition, distinguishing true homologous recombination from alignment artefacts or phylogenetic incongruence caused by reassortment remains challenging in highly diverse segmented RNA viruses such as CCHFV.

### 2.6 Statistical Tests

To assess potential differences in clinical manifestations between viral clades, we performed Wilcoxon rank‐sum tests (Mann–Whitney *U* test) for continuous variables and Fisher's exact tests for categorical variables [48, 49]. We analyzed 24 binary clinical parameters and 14 continuous biomarkers. Pearson correlation coefficients were calculated to examine relationships among 12 major biomedical measurements within each clade. *P*‐values < 0.05 were considered statistically significant for individual comparisons, though multiple testing corrections were applied where appropriate. A correlation coefficient with absolute values of 0.00–0.10 indicated a negligible correlation, 0.10–0.39 a weak correlation, 0.40–0.69 a moderate correlation, 0.70–0.89 a strong correlation, and 0.90–1.00 a very strong correlation [50]. All the statistical tests were performed using R [51], and correlations were visualized using the R package ggcorrplot [52].

## 3 Results

### 3.1 Phylogenetic Patterns and Multiple Reassortments of Co‐Circulating Variants in Sivas Province

**Figure 1:**
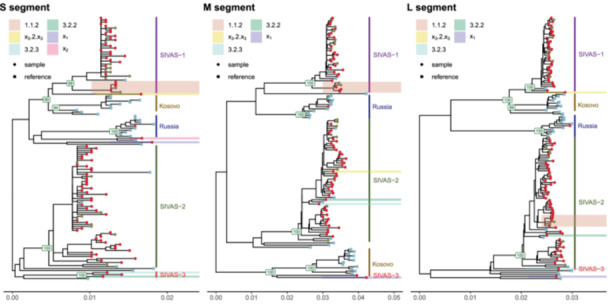
Reconstructed phylogenetic trees for each segment. Human samples (in brick red), tick samples (in gold), and reference sequences (in light blue) were used to reconstruct the phylogenetic trees of the S, M, and L segments, with 1649, 5336, and 12,122 nucleotides, separately. CCHFV variants in each segment tree were mostly clustered into five clades: SIVAS‐1, SIVAS‐2, SIVAS‐3, Russia, and Kosovo. Shades in different colors highlight the important differences between trees: red shade indicates the reassortment (1.1.2) of the L segment between SIVAS‐1 and SIVAS‐2; yellow shade indicates the reassortment ($x_{3}.2.x_{3}$) of the M segment between SIVAS‐2 and the unique strain $x_{3}$ in Sivas; cadetblue shade indicates the reassortment (3.2.3) of the M segment between SIVAS‐2 and SIVAS‐3; green shade indicates the reassortment (3.2.2) of the S segment between SIVAS‐2 and SIVAS‐3; and purple and pink shades indicate variants that do not belong to any clade ($x_{1},x_{2}$), *i.e*., found to be unique. Labels on nodes indicate the phylogenetic (bootstrap) support for the identified clades. The scale under each tree is in units of substitutions per site.

We analyzed complete tri‐partite genomes (S, M, and L segments) of CCHFV from 113 human infections and 23 ticks across the Sivas Province, a high‐prevalence region in Türkiye, and from one virus related to CCHFV in Russia. We identified three common clades shared across the S, M, and L segment trees (Figure 1); called SIVAS‐1, SIVAS‐2, and SIVAS‐3. The support from $10^{2}$ bootstrap data replicates was strong for these phylogenetic clades (99%–100% for S and 100% for M and L) and other important clades (94%–100% identifying Kosovo and Russia clades). Among our samples, SIVAS‐2 was the most prevalent clade (59%), followed by SIVAS‐1 (36%), and the much less prevalent clade SIVAS‐3 (1.7%) (Table 1). Based on sampling dates, SIVAS‐1 and SIVAS‐2 have been circulating in Sivas for more than a decade. All our new Turkish CCHFV variants belong to the previously established Europe‐1 lineage [11, 53].

**Table 1:** Phylogenetic clade distribution of Turkish CCHFV genomes across segment trees.

	S segment	M segment	L segment
SIVAS‐1	37	34	37
SIVAS‐2	58	53	63
SIVAS‐3	3	1	1
unique variants	3	1	2
Russian clade	1	1	1
TOTAL	102	90	104

All three SIVAS clades were involved in reassortments, with the most prevalent reassortant form being between SIVAS‐1 in the S and M segments and SIVAS‐2 in the L segment (reassortment 1.1.2). The fact that the sequences from reassortant 1.1.2 clustered together in each segment tree indicates that this reassortment happened once and then spread to several different hosts. This reassortant form was detected in four human and two tick samples across the S and L segment trees, with three human and the same two tick samples in the M segment tree. Additional reassortments were observed between SIVAS‐2 and SIVAS‐3, as evidenced by reassortants 3.2.3 and 3.2.2. This shows that all three segments can reassort independently. In detail, the reassortment pattern 3.2.3 was found in one human sample (118‐DD_S25), which carried SIVAS‐3 in the S and L segments and SIVAS‐2 in the M segment. Reassortment pattern 3.2.2 was found in another human host (sample 5‐1_S21), which was SIVAS‐3 in the S segment and SIVAS‐2 in the M and L segments. Interestingly, one of our new variants (59_S39), denoted as unique variant $x_{2}$, clustered with a previously reported sequence (i.e., Turkey200310849, Accession no. DQ211649) in the S segment (Figure S1). However, Turkey200310849 clustered with 5‐1_S12 in SIVAS‐2 in segments M and L (Accession no. DQ211636, DQ211623), thus establishing another reassortant in Türkiye [14]. Finally, the M segment of SIVAS‐2 had also reassorted with another unique strain (denoted $x_{3}$, cladistically located outside the SIVAS‐1 clade in the S and L segments) observed in two human samples. Thus, overall, SIVAS‐2, the most common clade, was involved in all observed reassortments in our data (Figures 1 and S1).

Our analysis also revealed the broader genetic diversity present in Sivas, including two unique CCHFV variants ($x_{1}$ and $x_{2}$) found exclusively in human infections. These variants maintained consistent phylogenetic positions across all three segments and showed no evidence of recombination with the main local clades (SIVAS‐1, SIVAS‐2, SIVAS‐3) and other significant clades based on RIP analysis (Figure S3). The presence of these divergent lineages alongside the reassorting local clades suggests that while reassortment occurs between co‐circulating variants, completely distinct viral lineages can also establish infections without contributing to the local reassortment network.

Interestingly, we identified one human infection (26_S16) in Sivas that was related to previously‐sampled Russian CCHFV sequences (Figures 1 and S1). The Russian sequences, together with our Sivas case, formed a distinct clade in all three segments (100% bootstrap support). This sample came from an agricultural worker living in Yildizeli, Sivas, who had no history of travel to another city or abroad, making local tick acquisition the most parsimonious explanation. Two potential pathways could explain this lineage's introduction: (i) migratory birds transporting infected *Hyalomma* nymphs across long distances, or (ii) livestock trade bringing infected ticks to Yildizeli or neighboring districts. However, the specific introduction mechanism remains unknown. Thus, while this link to Russian CCHFV variants is phylogenetically clear, there is no known direct epidemiological link to Russia. Also noteworthy, and alleviating public health concerns, is that no Sivas sequences clustered with the previously‐identified Kosovo clade, known to be more pathogenic than other strains [3]. Importantly, our phylogenetic analysis showed that the Kosovo clade shared a most recent common ancestor with SIVAS‐1 in the S and L segments (99% and 100% bootstrap support, respectively) and with SIVAS‐3 in the M segment (100% bootstrap support) (Figures 1 and S1). This shows that the Kosovo clade, based on its discordant phylogenetic placement across our segment trees, is a reassortant of earlier, parental variants related to SIVAS‐1 in the S and L segments and SIVAS‐3 in the M segment.

Comparing our new full genome CCHFV sequences to 52 previously reported high‐quality full genome sequences showed that the SIVAS‐1 and 2 clades, as well as the reassortants and unique variants we describe here, were as of now only found in Türkiye (Figure S3). This full genome analysis also showed that in addition to previous sequences from Kosovo and Russia, a set of sequences from Armenia sampled in 2023 also belonged to this Turkish super‐clade [25, 43, 47], which is well separated from African and Asian full CCHFV genomes (Figure S2). As has been shown previously, there were also a few very divergent CCHFV variants in Türkiye related to sequences from Greece, forming a clade historically called Europe‐2 [53]. Overall, this full genome analysis suggests that CCHFV spread is mostly limited to nearby geographical regions.

### 3.2 Limited Geographic and Temporal Spread of Co‐Circulating Variants

**Figure 2:**
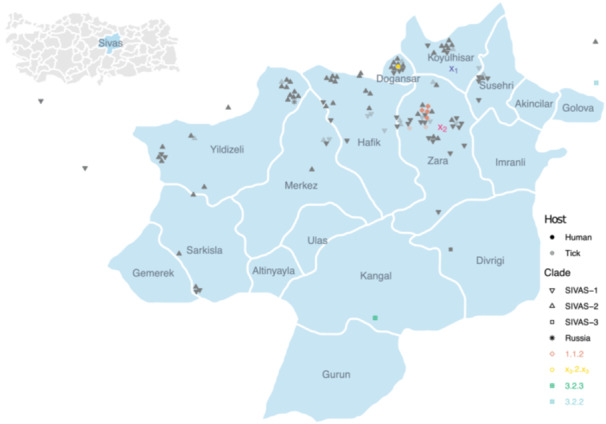
Geographic distribution of samples. Samples were collected in the Sivas region, in the middle of Türkiye. Human samples are in solid color, and tick samples are transparent. Interesting patterns are highlighted in colors: red diamonds indicate the reassortment (1.1.2) of the L segment between SIVAS‐1 and SIVAS‐2; yellow dot indicates the reassortment ($x_{3}.2.x_{3}$) of the M segment between SIVAS‐2 and unique strains in Sivas; square plus indicates reassortment (3.2.3) of the M segment between SIVAS‐2 and SIVAS‐3; and square cross indicates reassortment (3.2.2) of the S segment between SIVAS‐2 and SIVAS‐3. $x_{1}$ and $x_{2}$ on the map are unique CCHFV variants 1 and 2. Note that a few samples are from outside the Sivas region.

To assess possible spread patterns and associations between location and reassortment, we mapped sampling locations and labeled them according to their phylogenetic classifications. Sampling of CCHFV from humans and ticks occurred mostly in the northeastern districts of Sivas, with the highest densities reported in Yildizeli, Hafik, Dogansar, Zara, Koyulhisar, and Susehri (Figure 2).

Overall, SIVAS‐1 and SIVAS‐2 were detected across Sivas in several districts. On the contrary, the reassorted CCHFV forms (1.1.2, 3.2.3, 3.2.2, and $x_{3}.2.x_{3}$) were distributed sparsely, as were the unique variants $x_{1}$ and $x_{2}$.

While both SIVAS‐1 and SIVAS‐2 occurred in several districts, the most commonly observed reassortant (1.1.2) was sampled only in Zara, one of the districts with abundant samples, from both humans and ticks during the period of observation (2019, 2021, 2022, and 2023). This may suggest that the 1.1.2 reassortment occurred locally in Zara, although undetected circulation in nearby districts cannot be excluded due to incomplete geographic sampling. We also note that sampling sparsity in other districts may result in confined unique variants.

Both SIVAS‐1 and SIVAS‐2 were sampled from ticks and humans; while SIVAS‐1 was sampled during 2016–2023, and SIVAS‐2 was sampled over a somewhat longer period, 2011–2023, combining our new data with previously‐reported data. SIVAS‐3 was sampled in a human in Kangal in 2011. As for the reassortant form 3.2.3, it was sampled from a human in Kangal in 2021, and form 3.2.2 came from a human outside Sivas (in Giresun in 2021). As no other samples were available from those locations, we could not assess their possible origins.

Reassortant form $x_{3}.2.x_{3}$ was sampled in Dogansar in 2021. Interestingly, a previously‐reported sample from Sivas (TR‐SIVAS‐C13F, Accession no. ON668035, ON640906, ON191018), also sampled in 2021, is genetically close to our new sequence in all segments. The unique variants $x_{1}$ and $x_{2}$ were sampled in Koyulhisar in 2022 and Zara in 2016, respectively. The imported Russian form was sampled in the district of Yildizeli.

### 3.3 Assessment of Disease Severity of Main CCHFV Clades (SIVAS‐1 and SIVAS‐2) and the 1.1.2 Reassortant

To assess whether infections with different phylogenetic clades presented distinct clinical symptoms, we compared SIVAS‐1 with SIVAS‐2, for which we had the most data, using 24 binary clinical parameters and 14 continuous blood biomarkers. Human samples clustered into the same clade across all three segments were selected, yielding 14 from SIVAS‐1 and 37 from SIVAS‐2. The statistical test results are summarized in Table 2 and Table 3.

**Table 2:** Comparison of clinical symptoms, signs, and outcome between human CCHFV clade SIVAS‐1 and SIVAS‐2 infections (*n* = 51).

Variable	SIVAS‐1 (*n* = 14)	SIVAS‐2 (*n* = 37)	*p*‐value^a^
Symptoms, *n* (%)			
History of fever	11 (78.6)	33 (89.2)	0.376
Myalgia	14 (100)	36 (97.3)	1.000
Weakness	14 (100)	35 (94.6)	1.000
Headache	8 (57.1)	30 (81.1)	0.147
Nausea and/or vomiting	7 (50)	30 (81.1)	0.038**
Diarrhea	6 (42.9)	14 (37.8)	0.758
Sweating	8 (57.1)	16 (43.2)	0.531
Signs, *n* (%)			
Fever, $>\;38^{\circ }C$	11 (78.6)	29 (78.4)	1.000
Tachycardia, >100 bpm	5 (35.7)	18 (48.6)	0.533
Hypotension, TA <90∕60 mmHg	2 (14.3)	4 (10.8)	0.661
Maculo‐papular rash	6 (42.9)	16 (43.2)	1.000
Petechia/Ecchymosis	6 (42.9)	11 (29.7)	0.508
Epistaxis	4 (28.6)	9 (24.3)	0.734
Hematemesis	0 (0)	3 (8.1)	0.552
Melena	4 (28.6)	3 (8.1)	0.080*
Hematuria	3 (21.4)	4 (10.8)	0.376
Hemoptysis	0 (0)	2 (5.4)	1.000
Jaundice	0 (0)	3 (8.1)	0.552
Hepatomegalia	0 (0)	5 (13.5)	0.305
Splenomegalia	8 (57.1)	21 (56.8)	1.000
Bleeding	4 (28.6)	9 (25)	1.000
Confusion	2 (14.3)	4 (10.8)	0.661
Fatal outcome, *n* (%)	1 (7.1)	1 (2.7)	0.478
ICU admission, *n* (%)	1 (7.1)	3 (8.1)	1.000

^a^ p‐values according to Fisher's exact test (* *p* ≤ 0.1; ** *p* ≤ 0.05).

**Table 3:** Comparison of biochemical measurements and clinical scoring between human CCHFV clade SIVAS‐1 and SIVAS‐2 infections (*n* = 51).

Variable	SIVAS‐1 (*n* = 14)	SIVAS‐2 (*n* = 37)	p‐value^a^	Normal
Blood routine				
WBC (1$\;\times \;10^{3}∕{\;\text{mm}\;}^{3}$)	3.4 (2–4.8)	2.5 (1.9–3.5)	0.173	4–10.5
PLT (1$\;\times \;10^{3}∕{\;\text{mm}\;}^{3}$)	79 (48.8–105)	102 (76–123)	0.157	150–450
RBC (1$\;\times \;10^{6}∕{\;\text{mm}\;}^{3}$)	4.9 (4.7–5.3)	4.8 (4.5–5.2)	0.486	4.3–5.9 (male)
				3.5–5.5 (female)
Blood biochemistry				
AST (IU/L)	65.5 (39.5–134.5)	41 (30–121)	0.650	(0–40.0)
ALT (IU/L)	35 (16.2–50.5)	27 (22–77)	0.460	(0–41.0)
CPK ($\mu {\;\text{g/mm}\;}^{3}$)	350 (126–758)	175.5 (116.2–439.8)	0.292	(0–170.0)
LDH (IU/L)	400 (267.8–608.2)	289 (224–442)	0.217	(135–214)
Coagulation function				
PT (second)	13.3 (10.9–14.1)	12.1 (11.5–14)	0.735	(9.8–12.1)
aPTT (second)	30.6 (26.6–41.2)	32 (27.1–37.7)	0.760	(22.1–28.1)
fibrinogen (mg/dL)	233.7 (210.9–319)	259 (227.7–306)	0.720	(18–350)
d‐dimer (mg/L FEU)	1.3 (1.2–23.1)	1.3 (0.8–2.7)	0.116	(0–0.55)
Severity score, median (IQR)				
DIC score	3 (2.2–4.8)	2 (1–3)	0.088*	N/A
SGS score	2 (1–5.8)	2 (1–4)	0.592	N/A
Length of Hospital Stay (days)	8 (7.2–8.8)	7 (6–9)	0.495	N/A

*Note:* Data are presented as median (IQR) after eliminating missing data. SG Score mortality risk, High risk (10–16 points), Intermediate risk (5–9 points), Low risk (0–4 points).

^a^ p‐values according to the Wilcoxon rank‐sum test (* *p* ≤ 0.1).

Abbreviations: ALT, alanine aminotransferase; aPTT, activated partial thromboplastin time; AST, aspartate aminotransferase; CPK, creatine phosphokinase; DIC Score, Disseminated Intravascular Coagulation Score; FEU, Fibrinogen Equivalent Units; LDH, lactate dehydrogenase; PLT, platelets; PT, prothrombin time; RBC, red blood cell/erythrocyte; SG Score, Severity Grading Score; WBC, white blood cell.

Case‐fatality rates (fatal outcome) were 7.1% for SIVAS‐1% and 2.7% for SIVAS‐2 (p=0.478, Fisher's exact test). For the 1.1.2 reassortant, we had only three human infections (two from ticks), with no deaths among the infected individuals. Thus, we found no significant difference in mortality between SIVAS‐1 and SIVAS‐2, and at this time, no apparent elevated mortality rate in the SIVAS‐1/SIVAS‐2 (1.1.2) reassortant.

Among clinical symptoms and signs, only ‘Nausea and/or vomiting’ showed a statistically significant difference (p=0.038<0.05, Fisher's exact test), while Melena showed a borderline difference (p=0.080<0.1). For continuous biomarkers, the DIC Score showed a borderline difference (p=0.088<0.1, Wilcoxon rank‐sum test). However, after Bonferroni correction for multiple comparisons, none of these differences remained statistically significant, given 38 tests in total. No temporal trends of clinical parameters or blood biomarkers were seen.

**Figure 3:**
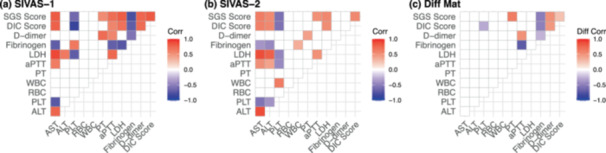
Correlation between numeric biochemical measurements in SIVAS‐1 and SIVAS‐2. The Pearson correlations between 12 continuous measurements are computed for clades (a) SIVAS‐1 and (b) SIVAS‐2, respectively. By subtracting two correlation matrices, we have the (c) Diff Mat, showing how different the correlation patterns are between SIVAS‐1 and SIVAS‐2 clades. Only significant (*p* < 0.05) correlations/differences are shown.

While no clinical parameters or biomarkers were significantly different between SIVAS‐1 and SIVAS‐2 infections, some trends in inter‐biomarker correlations could be observed (Figure 3). For SIVAS‐1, fibrinogen showed significant negative correlations with aPTT, LDH, D‐dimer, DIC Score, and SGS Score, while fibrinogen showed no significant correlation with these parameters in SIVAS‐2. Comparing the two‐parameter correlations between SIVAS‐1 and SIVAS‐2 infections revealed significant differences in some interactions. For instance, the positive correlation between SGS Score and PT and the negative correlation between ‘fibrinogen’ and ‘aPTT’ were significantly different in SIVAS‐1 (Figure 3C). The differences also suggested that fibrinogen correlated with SGS Score, DIC Score, and D‐dimer were lower in SIVAS‐1 infected patients.

## 4 Discussion

In this study, we have assessed the CCHFV spatio‐temporal genetic diversity in Sivas, a high‐prevalence, endemic region of Türkiye. We analyzed full‐genome sequences (S, M, and L segments) from both human and tick hosts. We found that although genetic diversity was high across the districts of Sivas, it was locally limited by geography. Adult *Hyalomma* ticks do not readily disperse without hosts; instead, long‐distance movement is primarily driven by nymphs attached to migratory birds and by movement of infested livestock. This ecology helps explain why we occasionally detect distant introductions, such as the sequence clustered in the Russian clade, yet observe limited onward spread within the province. Vertebrate amplification hosts may also contribute to reassortment opportunities by supporting transient co‐circulation of genetically distinct CCHFV strains, thereby enabling feeding ticks to acquire mixed infections within localized transmission cycles. Furthermore, we identified at least five reassorted CCHFV forms that appeared to have originated locally, where the parental forms were present, rather than spreading throughout the larger Sivas province. The repeated involvement of SIVAS‐2 in reassortment may reflect its greater local prevalence, increasing opportunities for mixed infection in ticks or vertebrate hosts. Alternatively, it may indicate differences in viral fitness, vector compatibility, or stochastic amplification within local transmission foci. Further sampling and experimental work would be required to distinguish between these possibilities. These results suggest that CCHFV mainly spreads slowly across geography, even though bird, livestock, and human hosts can travel fast and far. Indeed, we found one case in Sivas that phylogenetically clustered closely with Russian CCHFV forms. No further spread of this form was found in our study.

It has previously been shown that reassortment of genetically diverse Bunyaviruses can increase disease severity [25, 54] and that local endemic and phylogenetically unique forms of CCHFV may acquire higher virulence with significantly elevated case‐fatality rates. No CCHFV sequences from Sivas were close to the Kosovo variants, which have been reported to be more virulent with a 30–50% case‐fatality rate [55, 56]. Interestingly, with the establishment of the SIVAS‐1, 2, and 3 lineages in this study, we found evidence for the Kosovo clade to be a reassortant of early parental variants of SIVAS‐1 and 3, thus adding to the observation that reassortment may create more virulent strains. To investigate whether the most well‐represented clades in our data (SIVAS‐1 and SIVAS‐2) versus the 1.1.2 reassortant were more virulent, we compared their clinical data to each other. Consequently, comparing 38 clinical markers did not reveal any statistically significant differences in disease severity among these three groups. While no individual clinical parameters or biomarkers differed between SIVAS‐1 and SIVAS‐2 infections, we observed differences in biomarker‐to‐biomarker correlations. In particular, fibrinogen correlated with PT, SGS score, DIC score, and D‐dimer differed between SIVAS‐1 and SIVAS‐2 infections. It is unclear whether these differences are virus or host‐dependent.

Several limitations should be acknowledged. While our study, to the best of our knowledge, is the largest study of CCHFV in an endemic area that includes full‐genome sequence data from both tick and human hosts, our geographic and temporal sampling is still quite limited compared to the actual numbers of tick and human infections, which likely number in the thousands among humans during the period of observation (2011–2023). The limited human sample sizes for reassortant forms (n=3 for 1.1.2) restrict statistical power for clinical comparisons. Also, the human cases included were those who had reported to hospitals, possibly biasing the sample toward more severe cases. Nevertheless, we found robust evidence that both ticks and humans were infected with the same local variants, including reassortant forms. Detection of reassortants in ticks is particularly important because it supports maintenance within the enzootic transmission cycle, rather than representing transient reassortment arising only during acute human infection.

## 5 Conclusion

Our comprehensive analysis of CCHFV in Sivas Province, Türkiye, reveals important patterns of viral evolution and transmission. We identified three main phylogenetic clades (SIVAS‐1, SIVAS‐2, and SIVAS‐3) and at least four reassortant forms. Critically, reassortment appears to be rare and geographically localized, depending on the density of ticks infected with different co‐circulating clades. The most common reassortant form (1.1.2) was restricted to a single district (Zara), suggesting limited geographic spread despite the mobility of potential hosts.

Our clinical analysis comparing SIVAS‐1, SIVAS‐2, and their reassortant form (1.1.2) found no significant differences in disease severity and case fatality rates among these variants during our sampling period. This finding is reassuring from a public health perspective, as it suggests that reassortment between these common forms has not yet led to enhanced pathogenicity in humans.

However, the presence of multiple co‐circulating variants and ongoing reassortment highlights the need for continued surveillance. Future monitoring should focus on detecting novel reassortant forms and assessing their potential impact on disease severity. Enhanced surveillance, combining genomic sequencing with clinical data collection, will be essential for the early detection of variants with altered virulence characteristics and for informing public health responses to CCHFV outbreaks.

## Author Contributions

Conceptualization and project administration: Carmen Molina‐París, Thomas Leitner, Roger Hewson, Zati Vatansever, Nazif Elaldi. Study design: Carmen Molina‐París, Thomas Leitner, Roger Hewson, Zati Vatansever, Nazif Elaldi. Data collection and field work: Tuba Nur Tasseten, Nazif Elaldi, Binnur Koksal, Ayse Nur Pektas, Yasemin Cakir Kiymaz, Ahmet Deniz, Mesut Yigit, Hilal Bedir, Baris Yildiz, Zati Vatansever. Laboratory work: Mesut Yigit, Baris Yildiz, Ahmet Deniz, Zati Vatansever, Nazif Elaldi, Ayse Nur Pektas, Tuba Nur Tasseten, Yasemin Cakir Kiymaz. Bioinformatics: Baris Yildiz, Qianying Lin, Thomas Leitner. Formal phylogenetics and statistics: Qianying Lin, Thomas Leitner. Interpreted results: Qianying Lin, Thomas Leitner, Carmen Molina‐París, Martín López‐García, Grant Lythe, Roger Hewson, Nazif Elaldi, Zati Vatansever. Prepared first draft: Qianying Lin, Thomas Leitner, Carmen Molina‐París, Roger Hewson, Zati Vatansever, Nazif Elaldi. Edited the final manuscript: Qianying Lin, Thomas Leitner, Carmen Molina‐París, Roger Hewson, Zati Vatansever, Nazif Elaldi. All authors have read and approved the final draft of this manuscript.

## Conflicts of Interest

The authors declare no conflicts of interest.

## Supporting information

Supporting File

## Data Availability

The data that support the findings of this study are openly available in GenBank at https://www.ncbi.nlm.nih.gov/genbank/, reference number PX874843‐PX875138.
