# Supplementary material for: Multiple Reassortments, Limited Geographic Spread, and Comparable Pathogenicity of Crimean‐Congo Hemorrhagic Fever Viruses in Sivas Province, Türkiye
Source: J Med Virol. 2026 Jun 22;98(6):e71027. doi: 10.1002/jmv.71027 (PMC13284632; doi:10.1002/jmv.71027)
Supplement: Supplementary file 1 — Supporting File [file JMV-98-e71027-s001.pdf]

Multiple reassortments, limited geographic spread, and comparable pathogenicity of Crimean-Congo hemorrhagic fever viruses in Sivas Province, Türkiye

Supplementary Materials

Qianying Lin[1,2,†], Nazif Elaldi[3,†], Mesut Yigit[4,†], Baris Yildiz[5], Roger Hewson[6,7,8], Martín López-García[9], Grant Lythe[9], Ayse Nur Pektas[10], Binnur Koksal[11], Tuba Nur Tasseten[12], Ahmet Deniz[13], Hilal Bedir[13], Yasemin Cakir Kiymaz[3], Zati Vatansever[4,5], Carmen Molina-París[1,9,∗], Thomas Leitner[1,∗]

[1] *Theoretical Biology and Biophysics, Los Alamos National Laboratory, Los Alamos NM, USA*

[2] *Division of Biostatistics, The Ohio State University, Columbus OH, USA*

[3] *Department of Infectious Diseases and Clinical Microbiology, Cumhuriyet University, Sivas, Türkiye*

[4] *Department of Parasitology, Kafkas University, Kars, Türkiye*

[5] *Life Sciences and Technology Application and Research Center, Kafkas University, Kars, Türkiye*

[6] *Virology and Pathogenesis, UKHSA Porton Down, Salisbury SP4 0JG, UK*

[7] *Department of Infection Biology, London School of Hygiene and Tropical Medicine, London WC1E 7HT, UK*

[8] *Wellcome Sanger Institute, Hinxton CB10 1SA, UK*

[9] *School of Mathematics, University of Leeds, Leeds LS2 9JT, UK*

[10] *Cumhuriyet University Advanced Technology Application and Research Center (CUTAM), Cumhuriyet University, Sivas, Türkiye*

[11] *Department of Nutrition and Dietetics, Cumhuriyet University, Sivas, Türkiye*

[12] *Department of Molecular Biology and Genetics, Cumhuriyet University, Sivas, Türkiye*

[13] *Department of Parasitology, Faculty of Medicine, Kafkas University, Kars, Türkiye*

† *equal contribution*

∗ *correspondence:* tkl@lanl.gov *and* molina-paris@lanl.gov

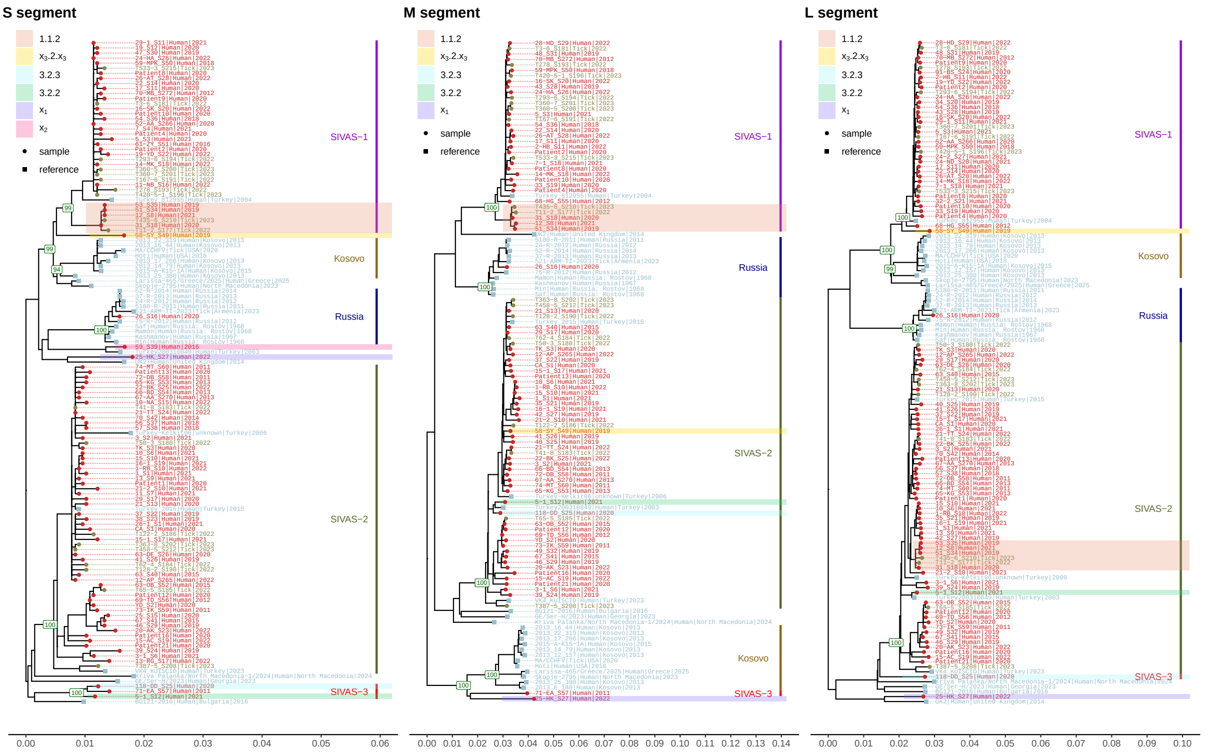

Fig. S1: **Reconstructed phylogenetic trees for each segment with labels.** Human samples (in brick red), tick samples (in gold), and reference sequences (in light blue) were used to reconstruct the phylogenetic trees of the S, M, and L segments, with 1,649, 5,336, and 12,122 nucleotides, separately. CCHFV variants in each segment tree were mostly clustered into five clades: SIVAS-1, SIVAS-2, SIVAS-3, Russia, and Kosovo. Shades in different colors highlight the important differences between trees: red shade indicates the reassortment (1.1.2) of the L segment between SIVAS-1 and SIVAS-2; yellow shade indicates the reassortment (x_3.2.x_3) of the M segment between SIVAS-2 and the unique strain x_3 in Sivas; cadetblue shade indicates the reassortment (3.2.3) of the M segment between SIVAS-2 and SIVAS-3; green shade indicates the reassortment (3.2.2) of the S segment between SIVAS-2 and SIVAS-3; and purple and pink shades indicate variants that do not belong to any clade (x_1, x_2), *i.e.,* found to be unique. Labels on nodes indicate the phylogenetic (bootstrap) support for the identified clades. The scale under each tree is in units of substitutions per site.

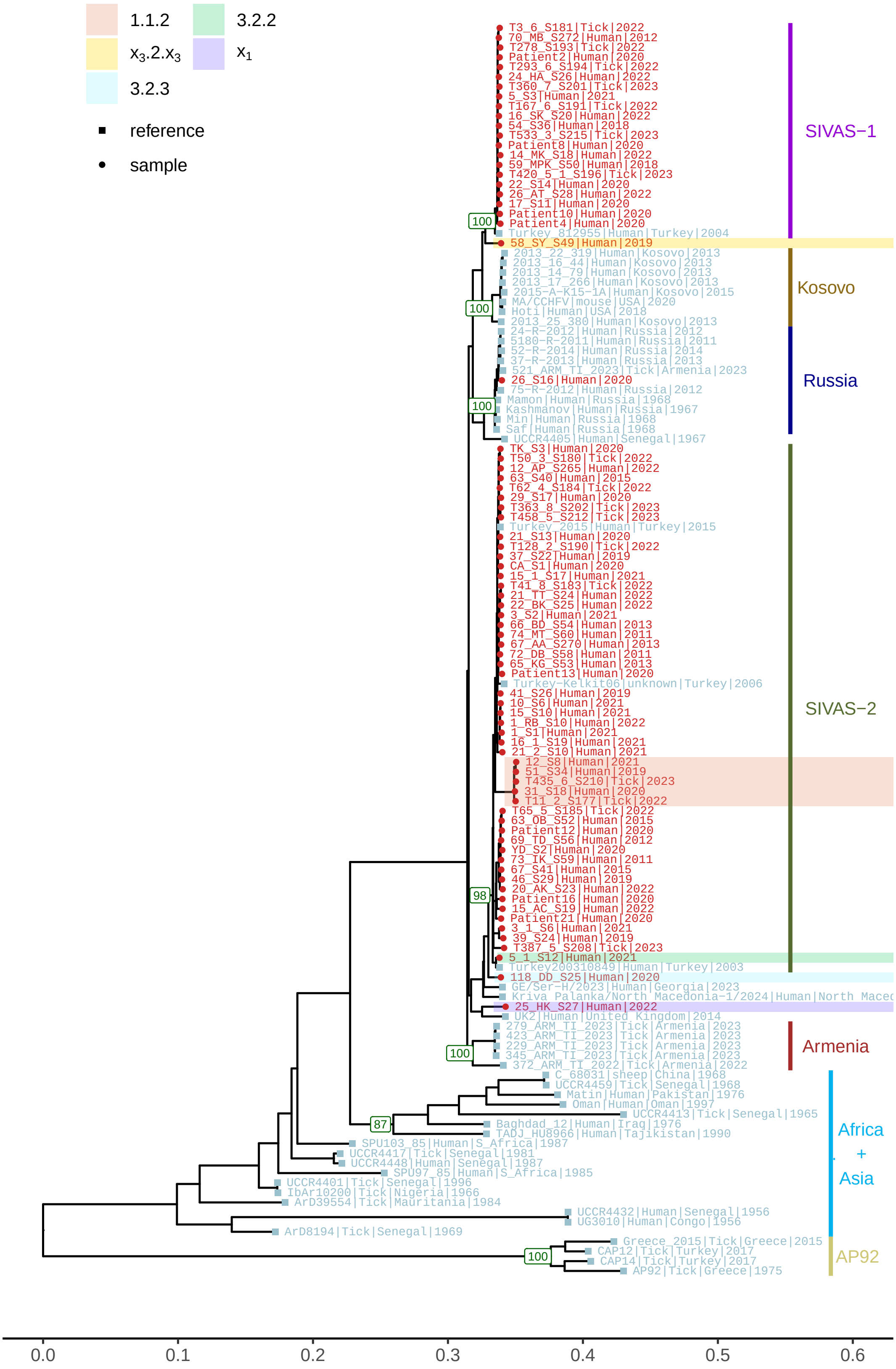

Fig. S2: The full-genome master tree reconstructed from concatenating S, M, and L segment sequences of samples and references. Colors and symbols are as in Fig. S1. Labels on nodes indicate the phylogenetic (bootstrap) support for the identified clades.

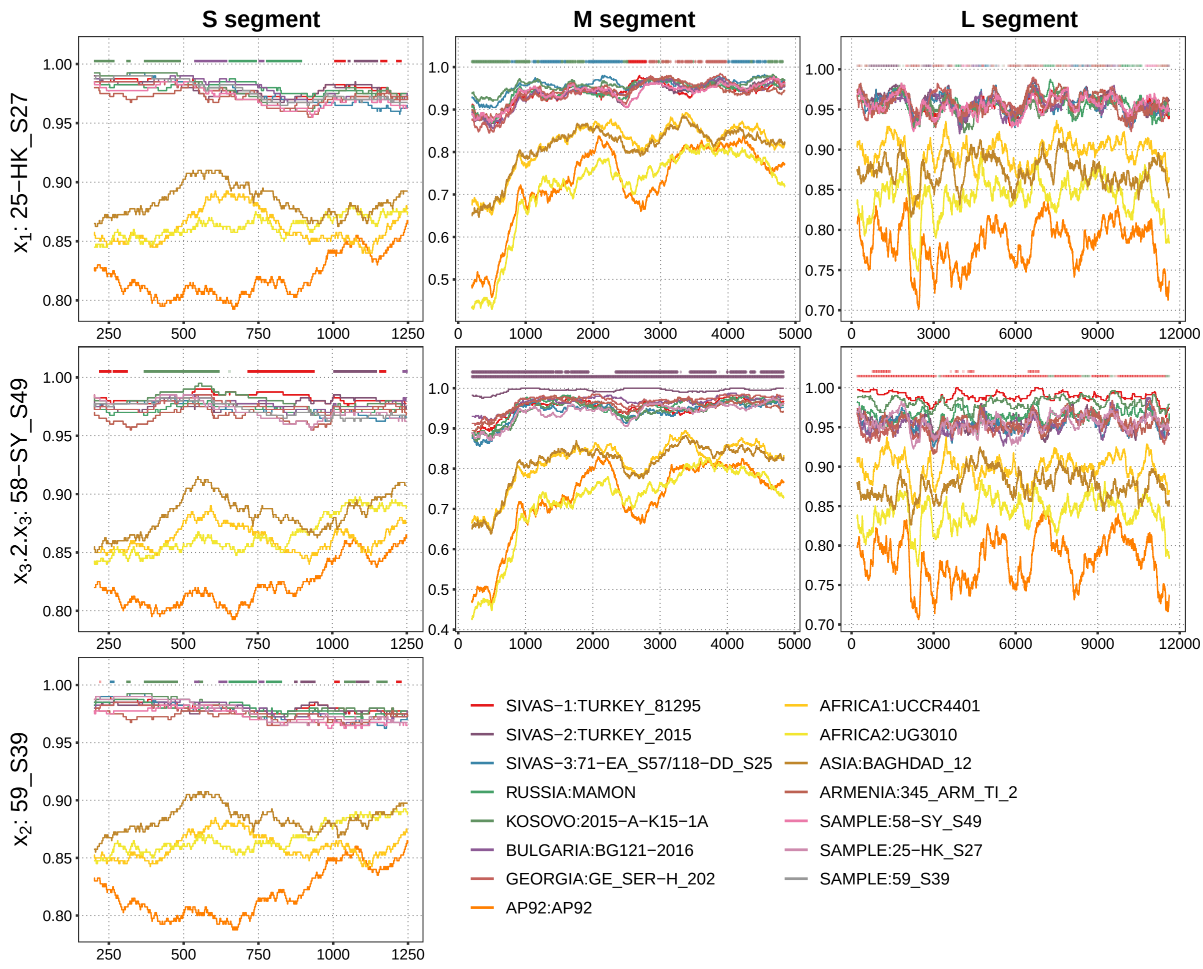

Fig. S3: RIP similarity scans for three CCHFV query sequences (rows) across the S, M, and L genome segments (columns). Each line shows the windowed nucleotide similarity $s(k)$ between the query and one reference sequence (legend) as a function of nucleotide position k, computed with a 400-nt sliding window. Colored dashes above $s = 1$ mark positions where a reference exceeded the 95% confidence threshold for the most similar reference at that window.
